# Evaluation of wooden spatula and plastic pipette methods for caries risk assessment in infants and toddlers

**DOI:** 10.1590/0103-644020256407

**Published:** 2025-04-14

**Authors:** Silvina Tineo, Paulo Nelson-Filho, Thais Citolino Barbosa, José Maria Alvarez Gimenez, Raquel Assed Bezzera da Silva, Lea Assed Bezzera da Silva, Marta Estela Saravia

**Affiliations:** 1 Department of Preventive Dentistry, School of Dentistry, National University of Tucumán, San Miguel de Tucuman, Tucumán, Argentina.; 2 Laboratory Oral Biology-LABOFOUNT, School of Dentistry, National University of Tucumán, San Miguel de Tucuman, Tucumán, Argentina; 3 Pediatric Dentistry Department, Ribeirão Preto School of Dentistry, University of São Paulo, Ribeirão Preto, São Paulo, Brazil

**Keywords:** Streptococcus, saliva, microbial, infant, dental caries

## Abstract

The study aimed to compare two different methods (wooden spatula and plastic pipette) for the collection of unstimulated saliva for colony counting of Mutans Streptococci species (MS) (microbiological caries risk), in infants and toddlers. The children’s behavior was favorable (very comfortable or comfortable) and unfavorable (uncomfortable or very uncomfortable), while the saliva collection, was also evaluated. Saliva samples were obtained from 19 children aged 1-29 months, of both sexes and seeded by both methods, obtaining the MS CFU numbers. The ANOVA test was used to statistically analyze the microbiological results, and the Z-test and chi-square test were used to analyze the behavioral assessment (α= 0,05%). 63.1% and 57.9% of children had MS in their saliva, using the saliva collection techniques with a spatula and a pipette, respectively. The number of CFUs was an average of 10.47 for saliva collected with the spatula and 7.32 for saliva collected with the pipette, however, there was no statistical difference between the methods (p=0.696653). Comparing the ages 1-6 months and 18-29 months, the older children showed higher CFU numbers, for both methods (p=0.000383). The clinical assessment of the child’s behavior demonstrated a significant statistical difference between the two methods, with more positive behavior for the spatula (p<0.001). In conclusion, the wooden spatula method can be used for saliva collection and quantifying of the MS levels in infants and toddlers, since there was no significant difference in the CFUs count, furthermore is better accepted based on the child's behavior, compared to the plastic pipette technique.

## Resumo

O estudo teve como objetivo comparar dois métodos diferentes (espátula de madeira e pipeta plástica) para coleta de saliva não estimulada para contagem de colônias de espécies de Estreptococos do grupo Mutans (EM) (risco microbiológico de cárie), em bebês e crianças de 1 a 3 anos. O comportamento das crianças, favorável (muito confortável ou confortável) e não favorável (desconfortável ou muito desconfortável), durante a coleta de saliva, também foi avaliado. Amostras de saliva foram obtidas de 19 crianças, de 1 a 29 meses de vida, de ambos os sexos, para obtenção das UFCs de EM. O teste ANOVA foi utilizado para a análise estatística dos resultados microbiológicos, e o teste-Z e o teste qui-quadrado foram utilizados para a análise da avaliação comportamental (α= 0,05%). 63,1% e 57,9% das crianças apresentaram EM na saliva, utilizando as técnicas de coleta de saliva com espátula e pipeta, respectivamente. O número de UFC foi em média 10,47 para a saliva coletada com espátula e 7,32 para a saliva coletada com a pipeta, porém sem diferença estatística entre os métodos (p=0,696653). Comparando as idades de 1 a 6 meses e de 18 a 29 meses, as crianças mais velhas apresentaram maior número de UFCs, para ambos os métodos (p=0,000383). A avaliação clínica do comportamento da criança demonstrou diferença estatística significativa entre os dois métodos, com comportamento mais favorável para a espátula (p<0,001). Concluindo, o método da espátula de madeira pode ser utilizado para a coleta de saliva e quantificação dos níveis de EM em bebês e crianças de 1 a 3 anos, uma vez que não houve diferença significativa na contagem de UFCs, além de ser mais bem aceito, com base no comportamento da criança, em comparação à técnica da pipeta plástica.

**Figure:**
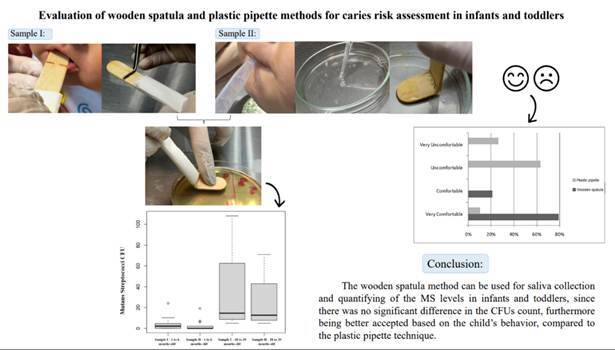

## Introduction

Early Childhood Caries (ECC) can be defined as the presence of one or more decayed (cavitated/non-cavitated lesions), missing, or filled surfaces due to caries in the primary teeth of children under six years old ^1^. This condition remains a significant chronic disease of childhood and a public health problem ^2^.

ECC is preventable, but currently affects more than 600 million children worldwide, causing a negative impact on children’s life quality. ECC, like other forms of dental caries, is considered to be a biofilm‐mediated, sugar‐driven, multifactorial, dynamic disease that results in the imbalance of demineralization and remineralization of dental hard tissues ^1^.

Bacterial colonization of an infant’s oral cavity is a key factor for caries risk ^3^. Traditional microbial risk markers for ECC include acidogenic-aciduric bacterial species, especially Mutans Streptococci (MS) species, that can be detected by microbial culture and molecular biology. It is known that high levels of *Streptococcus mutans* are a strong risk factor for ECC ^4^ and that the amount of *S. mutans* is significantly higher in the saliva and biofilm of children with cavitated lesions ^5^.

In children and neonates, saliva makes the perfect diagnostic medium for microbiological risk diagnosis of caries because of its noninvasive collection, easy handling, and storage of samples ^6^_._

There is a variety of saliva collection methodologies, but collection of unstimulated saliva by passive drool directly into plastic tubes ^7^ and sterile pipettes ^3^ are the most utilized methods. However, the choice of collection method should be carefully evaluated, considering the involved populations (elderly, neonates, and children) ^6^. According to Fey et al. (2024) ^8^, the absorption and suction methods, including the use of plastic pipettes, were preferred in situations involving young and uncooperative children.

In 1979, Köhler and Bratthall ^9^ developed a method (stamp method) to facilitate the estimation of MS levels in saliva, using wooden spatulas pressed directly against a selective medium, in adults and children under 3 to 6 years. However, the literature on this topic is scarce using this technique in young children ^10^_,_ and it has not been assessed so far in infants and toddlers (children aged 0 to 3 years old).

Thus, this study aimed to compare two methods (wooden spatula and plastic pipette) for the collection of unstimulated saliva for colony counting of MS (microbiological caries risk), in children 1-29 months old. The child’s behavior during the collection of saliva samples using the two techniques was also evaluated.

## Materials and methods

This study was carried out in the Oral Biology Laboratory of the School of Dentistry of the National University of Tucumán (LABOFOUNT) in Argentina, with samples obtained from infants and toddlers, of both sexes, from 1 to 29 months old (mean of 7.68 months). Children who used antimicrobial agents or antibiotics in the previous 3 months were excluded. The study was previously approved by the Research Ethics Committee involving Human Beings of the School of Medicine of the UNT (PIUNT J/611). The signing of the informed consent form was required before participation, by the mother, who signed on her name and her child.

**Figure 1:**
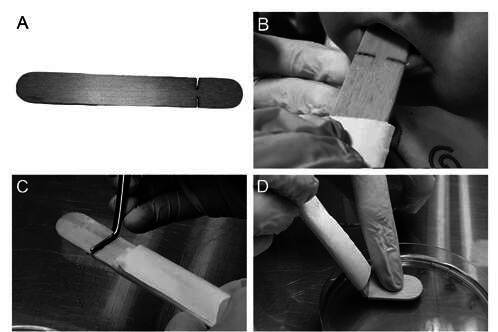
Collection of saliva samples in infants and toddlers, by wooden spatula technique and seeding in SB-20M culture medium. A - Adapted wooden spatula; B - Collection of saliva sample; C- Spatula breakage with the aid of sterilized tweezer; D - Sample seeding in the SB-20M culture medium.

Saliva samples were collected from each patient using 2 different methods (Sample I and Sample II), sequentially, in the morning, at least 2 hours after the last food intake and the last cleaning of the oral cavity:

Sample I (n=19): Unstimulated saliva samples were collected by the wooden spatula method ^9^. An adapted wooden sterilized spatula (Figure 1A), was introduced into the oral cavity of each child (Figure 1B), being moistened with saliva using rotating movements. It should be highlighted that the spatula was gently pressed not only on the tongue’s surface but also on all surfaces of the oral cavity, mainly on the cheek’s surface and on the back of the tongue, for 1 minute. Then, the spatula was broken with the aid of sterilized tweezers (Figure 1C) and softly pressed on a Petri dish containing the SB-20M culture médium ^11^ (Figure 1D).

**Figure 2:**
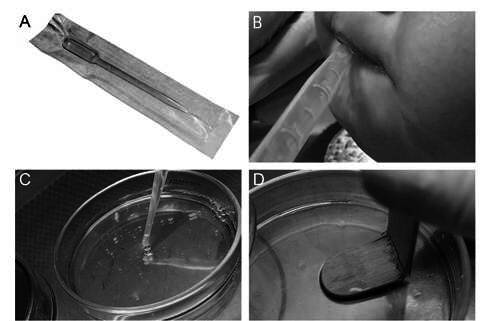
Collection of saliva samples in infants and toddlers, by disposable plastic pipette technique and seeding in SB-20M culture medium. A - Disposable plastic pipette; B - Collection of saliva sample; C- Saliva sample deposited in sterilized Petri dishes, for the immersion of the wooden spatula; D - Wooden spatula seeding in the SB-20M culture medium.

Sample II (n=19): Unstimulated saliva samples (2mL) were collected from the floor of the mouth with a 3mL disposable plastic pipette (Pasteur Prolab Materials for Laboratory Pipette - Brazil), by aspiration (Figures 2A and B). The 2mL of saliva was deposited in sterilized Petri dishes (Figure 2C). Wooden spatulas, similar to those used for sample I, were broken with the aid of sterilized tweezers and soaked in the saliva contained in these Petri dishes for 1 minute. Later, the spatula was softly pressed on a Petri dish containing the SB-20M culture medium ^11^, as described for sample I (Figure 2D). Thus, for each patient, the samples collected with the 2 techniques were seeded in the same Petri dish.

Subsequently, the samples I and II Petri dishes were incubated in anaerobic jars, under microaerophilic conditions, using the candle flame system, for 72 hours, at 37ºC ^12^ ^,^ ^13^. After the incubation period, MS Colony Forming Units (CFUs) were counted per mL of saliva, based on typical morphological characteristics. The count was carried out by an experienced evaluator (S.T.) and calibrated (*kappa*=0.85), using a stereomicroscope (ZTX-3D-C2 Numak, China). with reflected light, at 20x magnification, as described by Saravia et al. ^2^ ^,^ ^15^ ^) (^ ^12^. After counting the colonies the samples were divided into two groups based on the ages of the infants (1-6 months) and toddlers (18-29 months).

In cases of uncertainty about the morphological identification of MS, proteomic identification was performed by the MALDI-TOF mass spectrometry technique ^13^.

The clinical evaluation of infants' behavior during saliva sample collection for the wooden spatula technique and the plastic pipette technique, was also performed by an operator with clinical experience in Pediatric Dentistry (S.T.). The following categories were considered:

Very comfortable: The sample collection process was very comfortable for both the patient and operator.

Comfortable: The sample collection process was comfortable for both the patient and operator, with minor difficulty for the operator.

Uncomfortable: The sample collection process was not entirely comfortable for both the patient and the operator.

Very uncomfortable: The sample collection process was entirely uncomfortable for both the patient and the operator.

The microbiological results were statistically analyzed by the ANOVA, using the R language software. The Z-test using the R language and the chi-square test were used to analyze the behavior of the infants and toddlers. The significance level was set at 5%.

## Results

**Figure 3:**
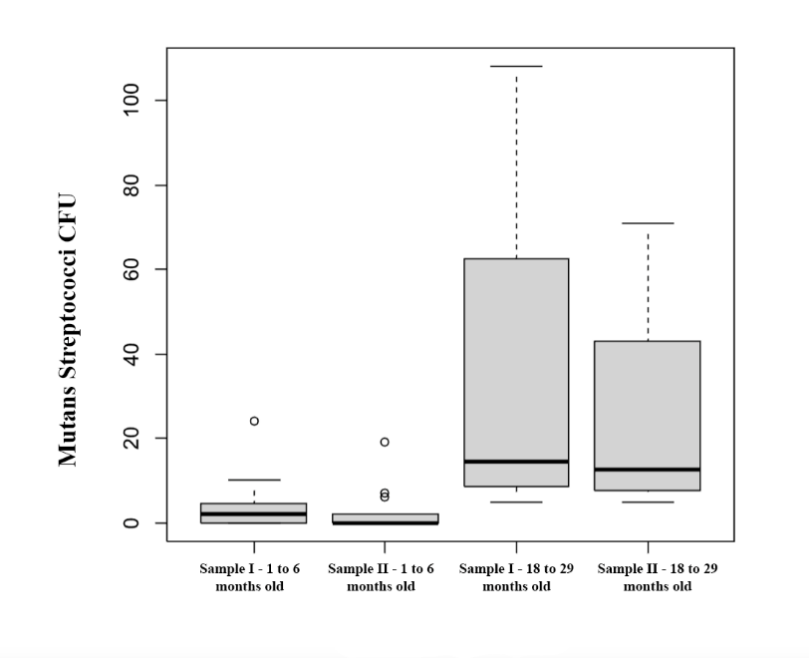
Boxplot showing the number of CFUs obtained in infants (children aged 1-6 months) and toddlers (children aged 18-29 months), after collecting saliva using a wooden spatula (Sample I) or plastic pipette (Sample II).

**Figure 4:**
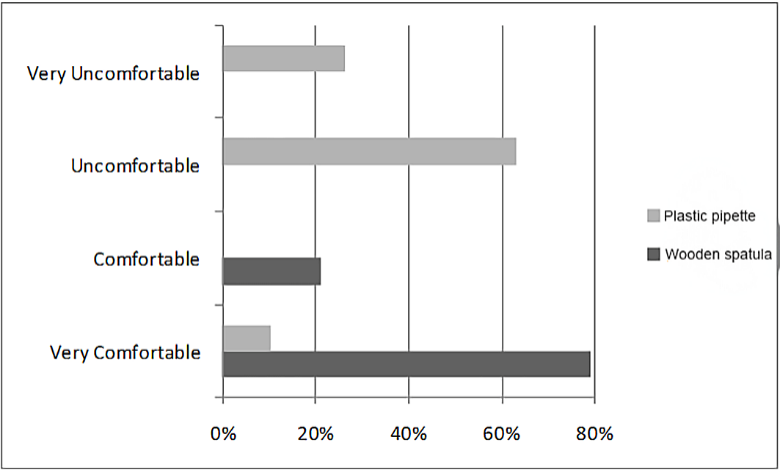
Results of the evaluation of infants and toddlers' behavior during unstimulated saliva collection using a wooden spatula or plastic pipette.

The results are expressed in Table 1 and Figures 3 and 4.

**Table 1:** Number of CFUs obtained after collecting unstimulated saliva using a wooden spatula or plastic pipette in infants and toddlers.

Patient	Age (months)	Sample I (wooden spatula)	Sample II (plastic pipette)
1	1	0	6
2	2	2	0
3	2	0	0
4	2	0	0
5	2	6	0
6	3	3	0
7	3	10	7
8	4	0	0
9	5	5	2
10	5	0	0
11	5	0	2
12	5	24	19
13	5	0	0
14	6	3	1
15	6	4	1
16	18	5	5
17	19	108	71
18	24	12	10
19	29	17	15
Total of CFU		199	137
Mean	7.68	10.47	7.32
SD	8.26	24.51	16.38
CV	107%	234%	224%

As shown in Table 1, it should be noted that MS was detected in the saliva of 63.1% and 57.9% of children, using the wooden spatula (Sample I) and the plastic pipette (Sample II) techniques, respectively. The number of CFUs ranged from 0 to 108, with an average of 10.47 for saliva collected with the wooden spatula and 7.32 for saliva collected with the plastic pipette. When comparing the two different methods of saliva collection in infants and toddlers, no statistically significant difference was observed between them (p=0.696653).

Figure 3 presents that in children aged 1-6 months, the average CFUs was 3.80 for the wooden spatula and 2.53 for the plastic pipette. On the other hand, in children aged 18-29 months, the average CFU was 35.50 for the wooden spatula and 25.25 for the plastic pipette. The statistical analysis showed a significant difference (p=0.000383) between the age groups of 1-6 months and 18-29 months, with higher numbers of CFU in older children, for both saliva collection techniques.

Figure 4 presents the results of the clinical assessment of the child’s behavior during the collection of saliva samples using the wooden spatula and plastic pipette techniques.

For the wooden spatula the predominant behavior was “Very Comfortable” (78.95%), while for the plastic pipette the predominant behavior was “Uncomfortable” (63.16%).

The statistical analysis showed a significant difference (p<0.001) between the saliva sample collection techniques, with positive behavior (very comfortable and comfortable) from the child when the wooden spatula was used.

## Discussion

According to Carrol (2024) ^14^, the factors found to be relevant in the development of ECC were the child's age, toothbrushing quality/plaque control, parental involvement in toothbrushing, fluoride exposure, pattern of dental attendance, dietary sugar exposure, habits, clinically carious active lesion presence, the socio-economic status of the child and their family, and the oral bacterial composition (*Streptococcus mutans* presence). Thang Le et al. (2021) ^15^ also observed that high levels of MS are a potential risk factor for ECC. However, obtaining saliva samples from young children (infants and toddlers) for microbiological caries risk assessment may present some difficulties related to cooperation and a disadvantageous perception of the procedure.

In this regard, during the clinical phase of the present study, we observed that obtaining saliva samples with the spatula, in this young population group, was easier for the operator and more convenient and comfortable for the child, compared to the plastic pipette. This fact shows that the wooden spatula technique can contribute to a more positive and less stressful experience during saliva collection. When using the pipette, it was clinically observed that the children closed their mouths more frequently and showed reflexes and actions of refusal during the saliva collection procedure. In parallel, the operator performed the procedure with a sense of unease, cautiously to avoid damaging the soft tissues, since the pipette has a rigid tip, unlike the wooden spatula which, despite being rigid, is circular and does not have sharp edges.

The collection of saliva samples in young children has been carried out mainly using sterile swabs ^16^ ^,^ ^17^, passive drool directly into sterile vials ^18^, sterile tongue blades ^19^ ^,^ ^20^, and pipettes ^3^. It should be noted that in studies that used sterile tongue blades, the samples were collected by keeping the spatula on the tongue for 1 minute. In the present study, we observed that children showed positive behavior when the wooden spatula was used, with a statistically significant difference, compared to the pipette technique. Possibly, the children accepted the procedure better with the spatula, as it was moved, for 1 minute, over all surfaces of the oral cavity, mainly the cheeks on both sides and the tongue, and not kept just on the surface of the tongue, which made the procedure clinically more pleasant for infants and toddlers.

The wooden spatula technique proposed by Köhler and Bratthall in 1979 ^9^ was initially described for adults and children under 3 to 6 years and used in different populations to determine caries risk, for example, in dental students ^12^ and children of up to 3 years old ^21^ ^,^ ^22^. Our results showed that this technique can be used to determine the microbiological caries risk in infants and toddlers. It should be noted that, due to the ease of implementation and the comfort it provides to the patient, the wooden spatula technique can also be used to quantify other microorganisms involved in pathologies of the oral cavity.

In the present study, two techniques for obtaining unstimulated saliva samples in children aged 1-29 months were compared, one by wetting a wooden spatula in the baby’s mouth and the other by absorbing saliva with a plastic pipette. The results with both techniques did not show a statistically significant difference in the CFU count of MS. As each child underwent saliva collection using both techniques, we can assume that possible differences would be attributed solely to the saliva sampling technique.

Another important result of the present study was the significant difference between the age groups of 1-6 months and 18-29 months, with higher numbers of CFUs in older children, for both the saliva collection techniques. These results are in agreement with those obtained by Fujiwara et al. (1991) ^23^, who evaluated the number and species distribution of salivary Mutans Streptococci in children aged 0-2 years old, observing that the detection rate of MS increased with age, correlated with the number of erupted teeth.

## Conclusion

Therefore, we conclude that the wooden spatula method can be used for saliva collection and quantifying of the Mutans Streptococci levels in infants and toddlers, since no significant difference was observed in the CFUs count, being better accepted based on the child’s behavior, as well as the operator clinically determined that using the spatula provides greater comfort, compared to the plastic pipette technique.
